# High-Resolution Melting PCR as Rapid Genotyping Tool for *Brucella* Species

**DOI:** 10.3390/microorganisms10020336

**Published:** 2022-02-01

**Authors:** Guillaume Girault, Ludivine Perrot, Virginie Mick, Claire Ponsart

**Affiliations:** EU-RL Brucellosis, Bacterial Zoonoses Unit, Animal Health Laboratory, University Paris-Est, ANSES, 94700 Maisons-Alfort, France; ludivine.perrot@anses.fr (L.P.); virginie.mick@anses.fr (V.M.); claire.ponsart@anses.fr (C.P.)

**Keywords:** *Brucella*, identification, SNPs, HRM-PCR

## Abstract

*Brucella* sp. are the causative agents of brucellosis. One of the main characteristics of the *Brucella* genus concerns its very high genetic homogeneity. To date, classical bacteriology typing is still considered as the gold standard assay for direct diagnosis of *Brucella*. Molecular approaches are routinely used for the identification of *Brucella* at the genus level. However, genotyping is more complex, and to date, no method exists to quickly assign a strain into species and biovar levels, and new approaches are required. Next generation sequencing (NGS) opened a new era into the diagnosis of bacterial diseases. In this study, we designed a high-resolution melting (HRM) method for the rapid screening of DNA and direct assignment into one of the 12 species of the *Brucella* genus. This method is based on 17 relevant single nucleotide polymorphisms (SNPs), identified and selected from a whole genome SNP (wgSNP) analysis based on 988 genomes (complete and drafts). These markers were tested against the collection of the European Reference Laboratory (EU-RL) for brucellosis (1440 DNAs extracted from *Brucella* strains). The results confirmed the reliability of the panel of 17 SNP markers, allowing the differentiation of each species of *Brucella* together with biovars 1, 2, and 3 of *B. suis* and vaccine strain Rev1 (*B. melitensis*) within 3 h, which is a considerable gain of time for brucellosis diagnosis. Therefore, this genotyping tool provides a new and quick alternative for *Brucella* identification based on SNPs with the HRM-PCR assay.

## 1. Introduction

Brucellosis, also known as ‘undulant fever’, is a zoonotic disease caused by Gram-negative bacteria of the *Brucella* genus [1]. Brucellae are a group of facultative intracellular Alphaproteobacteria that usually infect domesticated animals and humans, but also wildlife [1,2,3,4]. To date, the genus is composed of 12 species [5], mostly characterized by phenotypical and biochemical preferences: the six classical species *B. abortus* (bovine), *B. melitensis* (caprine and ovine), *B. suis* (porcine), *B. canis* (canine), *B. ovis* (ovine), *B. neotomae* (desert woodrat) [4,6,7,8], and six new species *B. ceti* (dolphins, porpoises and whales) [9], *B. pinnipedialis* (seals) [9], *B. microti* (common vole, frogs, wild boar) [10,11,12,13], *B. inopinata* (natural host not clearly identified, incidental association with breast implant) [14], *B. papionis* (baboon) [15] and *B. vulpis* [16]. The genus is therefore in constant evolution and new and atypical hosts are regularly identified, such as *Brucella* isolated in frogs [17,18] or more recently *Brucella* DNA detected in bats [19].

The population structure of the *Brucella* genus is highly clonal, with all *Brucella* species sharing sequence similarity values from 98% to 100% [20]. This particular genetic homogeneity explains the challenge of *Brucella* genotyping. Originally, the genus was classified as a single species subdivided into biovars [21]. In contrast to other bacteria, the ribosomal RNA gene sequences provide little information about internal separation within the genus as many members possess identical 16S rRNA sequences [22]. However, species and biovars can be differentiated using traditional microbiological tests, serological and phenotypic traits that match with the preferred host specificity [2,6]. These methods are considered tedious and hazardous for people in direct contact with live bacteria. The diagnosis of bacteria belonging to the *Brucella* genus was always difficult, especially concerning the biotyping of isolates and the gold standard approach is still the bacteriology [6,23]. Several specific high-resolution molecular methods are currently available for species identification, such as DNA-based methods (fragment analysis and sequencing) [2,20,24], restriction fragment length polymorphism PCR (RFLP) [25], multiple locus variable number of tandem repeat analysis (MLVA) [26,27], multilocus sequence typing (MLST) [28] or multilocus sequence analysis (MLSA) [29], SNP typing [3], average nucleotide identity (ANI) [30], and more recently the matrix-assisted laser desorption ionization-time of flight mass spectrometry analysis (MALDI-TOF) [31]. These molecular methods allow the distinction between most species of the *Brucella* genus, but to date, there is no perfect molecular method allowing to quickly and correctly assign a strain into the *Brucella* genus at species and biovar levels.

The WGS and the use of draft whole genome sequences in replacement of MLST and large-scale SNP analyses provide a powerful alternative to study the genome diversity [20]. Until now, hundreds of strains were sequenced and analyzed to produce some draft or complete genomes [32]. Some comparative studies of *Brucella* genomes demonstrated the high-resolution power of this kind of approach [2,3,33,34,35], as it was already established for other clonal bacterial species [36,37,38]. To date, more than 600 complete or draft *Brucella* genomes are available on NCBI and/or PATRIC. Based on these whole genome comparisons, it is possible to identify some specific phylogenetic variants that can be used within a genotyping tool, such as the HRM-PCR [37,39,40]. Previous studies on *Brucella* identification using HRM-PCR are reported, but not designed for the identification of all currently described *Brucella* species and associated biovars [41,42].

In this paper, we compared 988 *Brucella* genomes to identify 12,730 SNPs on the *Brucella* core-genome. A set of 17 fixed SNPs was identified in silico and used to develop a quick and accurate genotyping tool based on the HRM-PCR. This set of SNPs was tested and validated on a total of 1440 DNAs from different *Brucella* species. This genotyping tool will be used as routine tool in the network of EU Reference Laboratories for a quick *Brucella* identification.

## 2. Materials and Methods

### 2.1. wgSNP Analyses

Complete and draft genomes belonging to the *Brucella* genus were retrieved from NCBI (https://www.ncbi.nlm.nih.gov/genome/?term=brucella, 27 January 2022) and PAthosystems Resource Integration Center (PATRIC) (https://www.patricbrc.org/search/?keyword(brucella), 27 January 2022) [43]. A total of 988 genomes sequences (410 *B. abortus*, 370 *B. melitensis*, 79 *B. suis*, 49 *B.* sp., 35 *B. canis*, 17 *B. ovis*, 10 *B. ceti*, 6 *B. pinnipedialis*, 4 *B. neotomae*, 3 *B. microti*, 2 *B. vulpis*, 2 *B. papionis* and 1 *B. inopinata*) were used in this study (Supplementary File S1).

The genome of *B. melitensis* strain 16M was used as the reference genome for all the analyses. Chimeric genomes of chromosomes 1 and 2 were generated to compare complete and drafts genomes. Sequence analyses were performed in BioNumerics 7.6.2 (Applied Maths, BioMérieux, Marcy–l’Étoile, France). Synthetic sequencing reads of 250 bp and 50× coverage were generated in silico for all the sequences using ART [44]. The generated reads were mapped against the reference chimeric genome using BWA in BioNumerics. A set of SNPs was deduced for each genome sequence data using BioNumerics wgSNP module. Data were filtered by different ways: inter-SNP distance (minimum 10 bp between SNPs), repetitive elements (VNTR, rRNA), absolute coverage of 20× for each SNPs, unreliable bases (remove positions with at least one unreliable base, i.e., Ns), ambiguous bases (remove positions with at least one ambiguous base, i.e., IUPAC code) and gaps are removed. The matrix of filtered SNPs is used to generate a phylogenetic tree, using a maximum-likelihood approach (BioNumerics 7.6.2 and MEGA X), allowing phylogenetic analyses. The tree was rooted using strains of ‘atypical’ *Brucella*, as in a recent study [5]. Based on this phylogenetic tree, fixed variants specific to species and biovars are extracted and are used to develop the HRM-PCR assay.

### 2.2. DNA

DNA used in this study was extracted from pure bacterial suspension, either using phenol-chloroform for reference strains or High Pure PCR Template Preparation Kit (Roche^®^) for field strains. In total, 29 reference strains and 1411 field strains were used in this study.

### 2.3. HRM-PCR

High-resolution melting (HRM) is a post-PCR technique that determines with high precision the melt profile of PCR products. A new generation dye is incorporated into a double-stranded DNA. Using a slow constant increase in temperature, fluorescence acquisition allows the distinction between two different populations of amplicons. The method can be used to interrogate small number of SNPs. The filtered SNP matrix is composed of relevant variants. Species- or biovar-specific SNPs, i.e., having the specific variant, which is different from all other sequences, are extracted from this matrix. Once the fixed variants are identified, the 100 bp SNP flanking region is extracted, and primers are designed (Table 1) using Primer3plus (http://www.bioinformatics.nl/cgi-bin/primer3plus/primer3plus.cgi, 27 January 2022). A maximum amplicon size of 100 bp is expected, but most of the time, smaller amplicons are targeted.

Amplification was performed on the ViiA7™ Real-Time PCR System (Life Technologies, Carlsbad, CA, USA) using the LightCycler^®^ 480 High Resolution Melting Master Mix (Roche Diagnostics). The reaction mixture consisted of 0.2 μM of each primer, 1×LightCycler^®^ 480 HRM master mix and 2.5 mM MgCl2 in a 10-μL final volume. The following parameters were used: 10 min at 95 °C were followed by 40 cycles consisting of 10 s at 95 °C, 10 s at 58 °C and 20 s at 72 °C. Samples were next heated to 95 °C for 30 s, cooled down to 65 °C for 1 min and heated from 65 °C to 94 °C at a rate of 1 °C/s with 25 acquisitions/°C. For each HRM assay, a positive expected allele sample is always added, to compare the tested DNAs. The presence of non-specific amplicons was tested with water sample. The melting temperature and melting curves profiles were analyzed by the QuantStudio™ Real-Time PCR Software (version 1.2).

### 2.4. Assessment of HRM-PCR Performances

The HRM-PCR assay was designed to identify the species and biovars after the confirmation of the *Brucella* genus. The assay was challenged on intra-*Brucella* inclusivity and exclusivity for each couple of primers. For the inclusivity test (i.e., belonging to a specific *Brucella* species), all primers were tested on field DNAs from various *Brucella* species (previously identified by bacteriology). For the exclusivity (i.e., belonging to another *Brucella* species), all primers were tested on DNAs from other *Brucella* species that the previously identified species. 

The reference strains for each species are described in Supplementary File S2. For the inclusivity tests, all field DNAs belonging to the expected species were included (from 0 to 589 DNAs, Table 2). For the exclusivity tests, a randomly chosen panel was tested for each couple of primers (from 42 to 297 DNAs, Table 3), to avoid testing more than thousands of DNAs for each couple of primers.

## 3. Results

### 3.1. SNP-Based Phylogeny of the Brucella Genus

**Figure 1:**
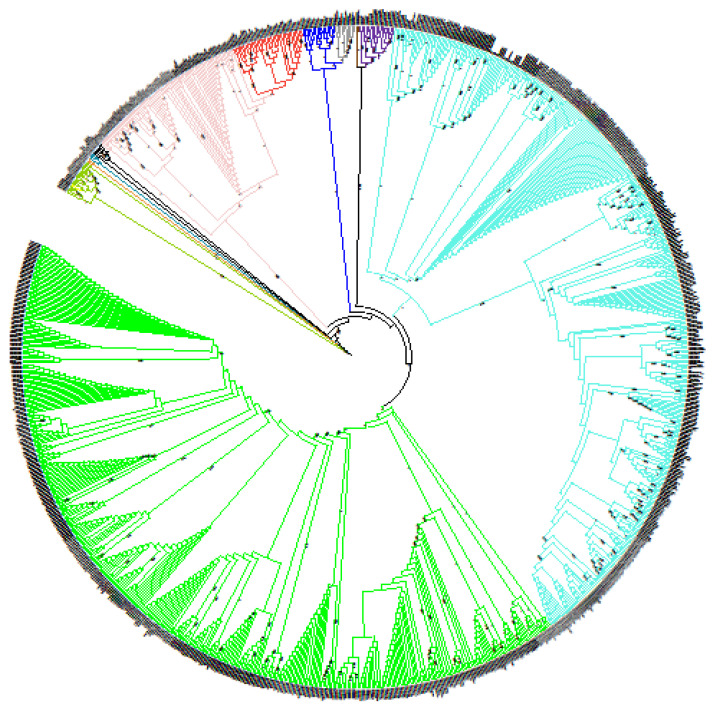
Global phylogeny of *Brucella* genus. Evolutionary history was inferred by using Maximum Likelihood method and Kimura 2-parameter model. Initial tree(s) for heuristic search was obtained automatically by applying Maximum Parsimony method. Tree is not drawn to scale (branch lengths does not correspond to number of substitutions per site). This analysis involved 988 genome sequences. There was a total of 12,730 positions in final dataset. Evolutionary analyses were conducted in MEGA X. All species are color-coded: green for *B. abortus*, light blue for *B. melitensis*, grey for *B. pinnipedialis*, dark blue for *B. ceti*, purple for *B. ovis*, brown for *B. papionis*, red for *B. canis*, pink for *B. suis*, black for *B. neotomae*, light green for *B*. sp. dark green for *B. microti*, yellow for *B. inopinata* and orange for *B. vulpis*. Bootstrap values are indicated at branch nodes (between 0 and 1).

A global comparison of complete and draft genomes of the *Brucella* genus generated by NGS was performed. Genome-wide comparison of 988 genomes yielded 12,730 SNPs after filtering allowing reconstruction of the *Brucella* genus phylogeny (Figure 1). This phylogenetic tree highlighted that all *Brucella* species were clearly separated into different clusters, i.e., each species can be defined by specific branches, thus providing a species-specific panel of SNPs. This phylogenetic tree was rooted with the clade of ‘atypical’ *Brucella* strains (including *B. inopinata* and strains isolated in bullfrogs) and as expected, *Brucella* species were split into two groups: the first one regrouping ‘classical species’ and the second group containing ‘atypical’ or early-diverging *Brucella* strains [5,45]. The most basal strain from the classical species was *B. microti* CCM 4915, whereas other lineages radiated quickly, indicating that all species diverged almost simultaneously, as previously reported [5,45].

### 3.2. Development of HRM-PCR Scheme

**Figure 2:**
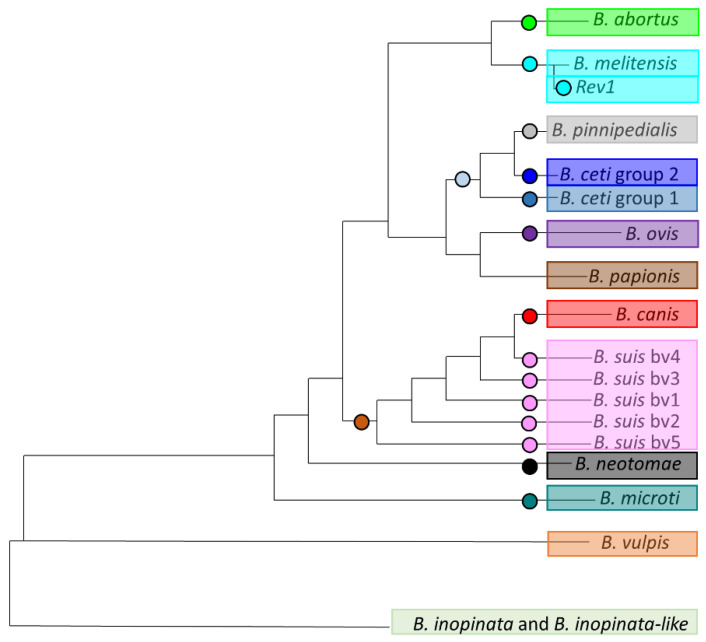
Position of 17 selected single nucleotide polymorphisms (SNPs) for high-resolution melting (HRM)-PCR on phylogenetic tree of *Brucella*. This figure represents a simplified phylogeny of *Brucella* genus. Each circle represent a fixed SNP selected on branch for identification of related species or biovar.

Once this phylogeny was well supported by NGS data, extracting fixed variants (SNPs) was the main crucial step to design HRM assays (Figure 2). A panel of 17 SNPs was thus selected, and specific primers were designed (Figure 2, Table 1). These SNPs were validated in silico for genotyping and the expected alternate alleles for different populations are indicated in Table 1.

### 3.3. Assessment of HRM-PCR Performances

These diagnostic assays were successfully validated on DNA extracted from reference strains for each species. A total of 29 reference DNAs were used and results were concordant in 100% of tests (inclusivity and exclusivity tests, Table 2 and Table 3). Results in Table 2 for reference DNA can be counted twice, as *B. suis* biovars DNA are included into the *B. suis*/*canis* primer test, explaining a higher number of DNA tested than 29. Finally, the developed HRM-PCR scheme was applied on the complete DNA collection of EU-RL. For the field collection, 1411 DNAs were tested, and results were concordant with previous bacteriological typing and multiplex PCR (Bruce-ladder and Suis-ladder) in 100% of cases (inclusivity and exclusivity tests, Table 2 and Table 3).

## 4. Discussion

Comparative whole-genome sequencing is a powerful and reliable way for characterization of any bacterial pathogen, especially for highly clonal bacteria like *B. anthracis*, *Brucella* sp., *Francisella tularensis* or *Mycobacterium tuberculosis* [37,38,45,46,47]. Cost, time, and biosecurity are important issues for all diagnostic assays. Concerning brucellosis, at least 10 days are required for bacteriology and classical phenotyping. In this aim, HRM-PCR is a very attractive assay, developed from WGS analyses, low-cost (only a couple of primers is required, approximately 0.5 € per test) and rapid. 

This study confirms that HRM-PCR can be applied to *Brucella* for a quick assignment of a DNA into a species. The results are very relevant for reference DNAs with 100% of results satisfying both inclusivity and exclusivity tests. Concerning field DNA, results are satisfying with 100% of concordant assignment for inclusivity and exclusivity tests. One important point about HRM-PCR concerns quality of DNA. In this study, a concentration between 10 and 100 fg was determined as the minimal concentration for DNA. This concentration was estimated using Qubit dsDNA High Sensitivity kit and serial dilutions of the DNA were performed and tested in PCR HRM. The last dilution detected was estimated at the minimal concentration of DNA required. This new HRM-PCR scheme is validated on reference strains (100% inclusivity and exclusivity) and on field samples (100% inclusivity and exclusivity). Relatively rare species like *B. vulpis*, *B. papionis,* or *B. inopinata* were not included in this scheme as they are not adequately represented in laboratories’ collection. Previous studies based on HRM-PCR allow the identification of five [41] to six species [42]. One of the main difficulties of the HRM-PCR remains the multiplexing of targets. A previous study allowed testing five targets in one multiplex reaction [41]. In this study, we decided to focus on the detection of all *Brucella* species without multiplexing, including identification of *B. suis* biovars. 

Using classic bacteriological typing takes almost 10 days to identify a strain. After DNA extraction, when using Bruce-ladder and Suis-ladder, results can be expected within 24 h. MALDI-TOF can provide results within a very short time and is mainly used for the identification of the genus, with remaining lacks of accuracy at species level [48]. Recent improvements were reported for *Brucella canis* species [49]. Nevertheless, the main bottleneck of MALDI-TOF is related to the database required to obtain a correct identification. The database should be well designed and enriched with spectra from different origins to correctly reach the species level. As regards other molecular methods, multiplex PCR (Bruce and Suis ladder) allows for the identification of species, vaccine strains, and biovars from *B. suis,* but the results are not obtained as fast as with HRM-PCR. With the panel of 17 markers designed in this study, it is possible to assign a DNA extracted from a strain into nine *Brucella* species (including all five biovars of *B. suis* and vaccine strain *B. melitensis* Rev1) in less than 3 h, which is an important gain in time in case of outbreak investigations. Moreover, even if DNA from reference strains is not available in all laboratories, the possibility to use synthetic amplicons (by oligonucleotides synthesis) as reference amplicon for each couple of primers is another advantage for this method. With the access to a thermocycler allowing HRM analysis, only the primers for the 17 SNPs assay, the synthetic amplicon used as reference profile and the DNA extracted from the suspected strain are necessary to identify precisely the species/biovars involved.

## 5. Conclusions

In this study, we propose a quick and easy-to-use way to assign whole genome data to a species using a molecular approach, directly developed from WGS data and wgSNP, the HRM-PCR. This genotyping tool developed in this study with the related 17 SNP panel dedicated to *Brucella* species and biovars is a very powerful approach to quickly identify a strain and assign it to the correct species. This method will be used in routine in EU-RL and can be applied mostly for brucellosis reference laboratories in the world. 

This SNP scheme will probably be improved in the future, as laboratories produce more and more data and share it with the scientific community. Moreover, the use of HRM-PCR directly on DNA extracted from specimens is a promising outlook, as preamplification step combined with HRM showed promising results. This will be the next step for future analyses, ‘filling the gaps’ to combat brucellosis.

## Figures and Tables

**Table 1:** SNPs used for HRM-PCR and related information.

Species Targeted	Genomic Position (16M ref)	Forward Primer (5′-3′)	Forward Primer Coordinates (16M ref)	Reverse Primer (5′-3′)	Reverse Primer Coordinates (16M ref)	Locus Tag	Locus Tag Information	Amplicon Size (bp)	Targeted Allele (Related to Column 1)	Other Allele (Related to All Other *Brucella)*
*B. abortus*	609866	acgaagaagcgatctcgatg	609832-609851	aggaaaggccgatgatgtaa	609905-609924	BMEI0587	coml, competence lipoprotein	93	T	C
*B. melitensis*	375209	cggtccgggccacctttacg	375164-375183	ggcccggcaattgctcctga	375225-375244	NR	NR	81	C	T
*B. suis-B. canis*	687223	ctggcggaaaaggatttgat	687162-687181	aatcacgacaaaccacagca	687232-687251	BMEI0664	sugar transport system permease protein	90	T	C
*B. suis* biovar 1	656162	tgacatggaccctgttttcc	656196-656215	cagcgtgacactgaacatgg	656138-656157	BMEI0629	hypothetical protein	78	G	A
*B. suis* biovar 2	221777	agaccttgcgcttgaacg	221821-221838	gccacactgctgagttcg	221755-221772	BMEI0215	(di)nucleoside polyphosphate hydrolase	84	T	C
*B. suis* biovar 3	1368151	gtatggcggaatgcagga	1368178-1368195	cacaaacgccagtgaacg	1368132-1368149	NR	NR	64	A	G
*B. suis* biovar 4	2026823	aagatcgccgtcgtctcg	2026873-2026890	ggccacaacagcctgaac	2026801-2026818	NR	NR	90	A	G
*B. suis* biovar 5	159143	cttccgttgaagggcaatc	159161-159179	gcctcgaaaacgaaatcatc	159085-159104	NR	NR	95	C	T
*B. canis*	937299	gagaactgacccgatggaaa	937238-937257	caagggaaccgaatatctgc	937302-937231	NR	NR	84	C	T
*B. microti*	1111504	aactgccggatgtgaaaaag	1111529-1111548	aaggatcgaggcgtcataaa	1111478-1111497	NR	NR	71	C	T
Marine *Brucella*	1237960	gcgatttcattgcccttg	1237892-1237909	ttgaaatgggcttcatcca	1237961-1237979	NR	NR	88	A	G
*B. ceti* 1	318627	aatgccgcaatcttcatctt	318637-318656	cctctgcgcgacagtttaag	318587-318606	NR	NR	70	A	C
*B. ceti* 2	121188	ctcgctcccaaacactaccc	121150-121169	cgttcgccccttatatttga	121220-121239	NR	NR	90	C	T
*B. pinnipedialis*	369804	tgcgggatttcaaggataag	369821-369840	aagatcgccagatcgtgct	369768-369786	BMEI0358	deoxyuridine 5′-triphosphate nucleotidohydrolase	73	T	C
*B. ovis*	576553	atgggctttggcggtatt	576495-576512	cgcccaggtagagctttg	576558-576575	BMEI0556	alpha-ketoglutarate permease	81	T	C
*B. neotomae*	1010822	atggcgaattcgatgaaaag	1010854-1010873	tgtcttcacagacgggaatg	1010775-1010794	NR	NR	99	T	G
*B. melitensis* Rev 1	139509	cttcacgccatgcttctttt	139556-139575	atgctcaccaccttcaacg	139483-139501	BMEI0141	dihydrolipoamide succinyltransferase component (e2) of 2-oxoglutarate dehydrogenase complex	93	T	C

**Table 2:** Results of inclusivity tests.

		Expected SNP Profile (Inclusivity Test)								
		Reference DNA				Field DNA				Total
		Expected Allele	Other Allele	Total	%	Expected Allele	Other Allele	Total	%	
Primers	*B. abortus*	11	0	11	100	96	0	96	100	107
	*B. melitensis*	6	0	6	100	232	0	232	100	238
	*B. melitensis* Rev1	2	0	2	100	6	0	6	100	8
	*B. suis/canis*	6	0	6	100	589	0	589	100	595
	*B. suis* biovar 1	1	0	1	100	26	0	26	100	27
	*B. suis* biovar 2	1	0	1	100	194	0	194	100	195
	*B. suis* biovar 3	1	0	1	100	2	0	2	100	3
	*B. suis* biovar 4	1	0	1	100	0	0	0	NA	1
	*B. suis* biovar 5	1	0	1	100	0	0	0	NA	1
	*B. canis*	1	0	1	100	82	0	82	100	83
	*B.* marine	3	0	3	100	14	0	14	100	17
	*B. ceti* group 1	1	0	1	100	8	0	8	100	9
	*B. ceti* group 2	1	0	1	100	4	0	4	100	5
	*B. pinnipedialis*	1	0	1	100	2	0	2	100	3
	*B. microti*	1	0	1	100	68	0	68	100	69
	*B. ovis*	1	0	1	100	88	0	88	100	89
	*B. neotomae*	1	0	1	100	0	0	0	NA	1
Total DNA				40				1411		1451

**Table 3:** Results of exclusivity tests.

		Other SNP Profile (Exclusion Test)								
		Reference DNA				Field DNA				Total
		Expected Allele	Other Allele	Total	%	Expected Allele	Other Allele	Total	%	
Primers	*B. abortus*	0	16	16	100	0	218	218	100	234
	*B. melitensis*	0	21	21	100	0	66	66	100	87
	*B. melitensis* Rev1	0	26	26	100	0	56	56	100	82
	*B. suis/canis*	0	22	22	100	0	43	43	100	65
	*B. suis* biovar 1	0	26	26	100	0	297	297	100	323
	*B. suis* biovar 2	0	25	25	100	0	63	63	100	88
	*B. suis* biovar 3	0	28	28	100	0	94	94	100	122
	*B. suis* biovar 4	0	25	25	100	0	52	52	100	77
	*B. suis* biovar 5	0	25	25	100	0	54	54	100	79
	*B. canis*	0	26	26	100	0	64	64	100	90
	*B.* marine	0	24	24	100	0	57	57	100	81
	*B. ceti* group 1	0	26	26	100	0	63	63	100	89
	*B. ceti* group 2	0	26	26	100	0	68	68	100	94
	*B. pinnipedialis*	0	26	26	100	0	70	70	100	96
	*B. microti*	0	26	26	100	0	59	59	100	85
	*B. ovis*	0	28	28	100	0	42	42	100	70
	*B. neotomae*	0	27	27	100	0	45	45	100	72

## Data Availability

Not applicable.

## Supplementary Materials

The following supporting information can be downloaded at: https://www.mdpi.com/article/10.3390/microorganisms10020336/s1, Supplementary File S1: List of genomes sequences used in this study; Supplementary File S2: list of reference DNA used in this study.
